# The effect of low-dose aspirin on aspirin triggered lipoxin, interleukin 1 beta, and prostaglandin E2 levels in periapical fluid: a double-blind randomized clinical trial

**DOI:** 10.1186/s12903-023-03243-0

**Published:** 2023-07-31

**Authors:** Elham Khoshbin, Razieh Salehi, Rooholah Behroozi, Soroush Sadr, Alireza Zamani, Maryam Farhadian, Hamed Karkehabadi

**Affiliations:** 1 Department of Endodontics, Dental School, Hamadan University of Medical Sciences, Hamadan, Iran; 2 Department of Endodontics, School of dentistry, Qazvin University of Medical Sciences, Qazvin, Iran; 3 Department of Immunology, School of Medicine, Hamadan University of Medical Sciences, Hamadan, Iran; 4 Department of Biostatistics, School of Public Health, Hamadan University of Medical Sciences, Hamadan, Iran; 5 Department of Endodontics, Dental Research Center, Hamadan University of Medical Sciences, Hamadan, Iran

**Keywords:** Specialized pro-resolving mediators, Low-dose aspirin, Aspirin triggered lipoxin, Prostaglandin E2, Interleukin 1 beta

## Abstract

**Background:**

The role of pro-resolving mediators in inflammation is a new concern in research. The effect of low-dose aspirin on production of a special kind of these mediators named aspirin triggered lipoxin (ATL) has been studied on different tissues. This randomized clinical trial evaluated the effect of low-dose aspirin on ATL and pro-inflammatory mediators’ level in periapical fluid of necrotic teeth with large lesions.

**Methods:**

Twenty-four patients with necrotic pulp and periapical lesion were randomly assigned to low-dose aspirin and placebo groups. In the first appointment, canals were shaped up to F3 size and #40 K-file and cleaned with 10 milliliters 2.5% sodium hypochlorite and 17% Ethylenediaminetetraacetic acid. Periapical fluid was sampled by a paper cone. The tooth was temporized without any intracanal medication. Tablets were administered for 7 days, then the teeth were re-opened and the sampling were repeated. Interleukin-1 beta (IL-1β), prostaglandin E2 (PGE2) and ATL were analyzed by enzyme-linked immunosorbent assay. Data were analyzed with paired t-test using SPSS statistical software, version 21 (α = 0.05).

**Results:**

A significant reduction in PGE2 and IL-1β was noted in the aspirin-treated group while an increase in ATL was observed (P < 0.001). There was no significant difference in the mediator scores before and after in the placebo-treated group (P > 0.05).

**Conclusion:**

Low-dose aspirin can influence the inflammatory process by reducing pro-inflammatory mediators such as PGE2 and IL-1β, as well as increasing the pro-resolving mediators such as ATL.

**Trial registration:**

IRCT20191211045702N1.

## Background

Apical periodontitis (AP) is a prevalent inflammatory lesion involving teeth caused by host response to endodontic infection that leads to periapical bone loss [1]. Bone resorption is a critical feature of periapical inflammatory lesions and is mediated by interleukin-1 beta (IL-1β), tumor necrosis factor-alpha (TNF-α) and prostaglandin E2- (PGE2) [2, 3]. At the onset of inflammation, these cytokines play an important role as pro-inflammatory agents in the development of chemotactic responses of leukocytes in the target tissue [4]. In the resolution phase, special pro-resolving lipid mediators (SPMs) are activated that actively limit inflammation and allow tissue repair [4]. The biological function of these mediators includes controlling PMNs, stimulating non-inflammatory cells, and increasing phagocytosis [5]. SPMs also regulate acute inflammatory cytokines (such as prostaglandins, leukotrienes, and other proinflammatory cytokines) and increase the production of anti-inflammatory mediators such as IL-10 [5, 6]. The realization of the application of SPMs and their unique ability to eliminate infection without any adverse side effects has led to extensive research to evaluate its therapeutic applications in the treatment of inflammatory diseases ranging from cardiovascular to periodontal disease [7]. These mediators are derived from unsaturated fatty acids and include lipoxins, maresins, resolvins, and protectins [8]. Lipoxins were first identified by Serhan et al. and they are produced during the enzymatic conversion of acid arachidonic in the lipoxygenase pathway [4].

Aspirin is a nonsteroidal anti-inflammatory drug (NSAID) and its anti-inflammatory properties has long been known [9, 10]. Its mechanism of action involves the irreversible blockade of both the cyclooxygenase-1 (COX-1) and cyclooxygenase-2 (COX-2) pathways. This blockade effectively stops the production of prostaglandins (PGs), which in turn leads to the reduction of T-cell activation [9, 10]. Recent studies shows that aspirin transforms COX-2 to a lipoxygenase-like enzyme which produces an epimer of lipoxin A4 which is termed 15-epi-lipoxin A4 or aspirin triggered lipoxin (ATL). ATL suppresses inflammation by inhibiting IL-1 and IL-6 expression [11]. Cells expressing COX-2 (including endothelial cells, epithelial cells, macrophages, and neutrophils) can produce ATL [12]. ATL formation has been shown in various studies on inflammation, including aspirin-sensitive asthma [13] and periodontitis [14]. Furthermore, it has been shown that aspirin can increase the production of ATL in periodontal tissues and it has positive clinical effects on pocket depth and clinical attachment level [14, 15].

Root canal treatment is the established therapeutic approach for treating AP by effectively reducing bacterial burden within the root canal system. However, root canal treatment exhibits a failure rate, reported to be as high as 20% [16]. Additionally, large periapical lesions often exhibit a cystic nature. These cystic lesions tend to be resistant to root canal treatment [17]. Consequently, there is a growing interest in exploring adjuvant immunomodulatory therapies to complement root canal treatment [2, 18, 19]. To the best of our knowledge, no study has investigated the anti-inflammatory and pro-resolving effects of aspirin in AP. Therefore, we investigated the effects of aspirin on the expression of IL-1, PGE2, and ATL in teeth with AP. The null hypothesis of this study was aspirin would not increase the levels of IL-1 and PGE2 and decrease the levels of ATL in teeth with AP.

## Materials and methods

The study was registered in Iranian registry of clinical trials (IRCT20191211045702N1) in 23/12/2019 and approved in the human research ethics committee in Hamadan dental school under protocol IR.UMSHA.REC.1398.692.

Patient selection: Patients referred to the endodontics department of Hamadan Dental School from January to March 2019 were examined and individuals with periapical radiolucent lesions resulting from a primary endodontic infection were selected.

Patients eligible for inclusion included those with good general health, patients between the ages of 18 and 45, single-rooted teeth, distal roots of mandibular molars and palatal roots of maxillary molars that had a periapical lesion of at least 5 millimeters in diameter, teeth which had no history of trauma or previous root canal treatment. Exclusion criteria were as follows: 1- Isolated non-treatable teeth, 2- Periodontal disease, 3- Presence of systemic diseases such as diabetes, hepatitis, bleeding disorder, autoimmune diseases, arthritis, AIDS, asthma, Kidney and liver disease, and hypertension, 4-history or presence of infections, 5-specific physical conditions such as pregnancy, lactation, and menstruation, 6-mouth ulcers, 7-regular smoking and alcohol consumption, 8- gastrointestinal disease such as ulcers 9- Treatment with any drug during the past week and 10- Allergy to salicylates or other NSAIDs, 11- patients receiving chemotherapy treatment.

The required sample size in each of the study groups was calculated based on the following formula:

$$n = \frac{{(\sigma _1^2 + \sigma _2^2){{({z_{1 - \frac{\alpha }{2}}} + {z_{1 - \beta }})}^2}}}{{{{({\mu _1} - {\mu _2})}^2}}}$$

The sample size calculation was based on values obtained from a prior study [20]. with a desired test reliability of 95% and a test power of 80%. Considering an expected difference of 60% and a standard deviation of 50%, the minimum required sample size was determined. Consequently, a total of 24 samples (12 samples per group) were deemed necessary to achieve adequate statistical power. The block randomization process, employing a block size of 4, was conducted by an individual independent of the main research team. Both the patients and physicians involved in the study were blinded to the group allocation of the individuals. Patients are randomly divided into case and control groups. To accurately assess the lesion, two examiners (an oral and maxillofacial radiologist and an endodontist) examine the x-rays taken from the patients. X-rays were taken using EzSensor and measurement was done using Ezdent-i software (Vatech, Hwaseong-si, South Korea). The purpose of the study and the effects of the drug for the patients were explained and the informed consent is signed by the volunteers. To diagnose the condition of the pulp, pulp sensitivity test is performed using cold spray and electric pulp tester.

Sampling of periapical fluid: First, the patient rinsed his/her mouth with 30 ml of 0.2% chlorhexidine mouthwash (Chloraxid, Iran najo, Tehran, Iran) for 30 s. After anesthetic injection of 2% lidocaine hydrochloride with 1.80,000 epinephrine (XYLOPEN, EXIR PHARMACEUTICAL CO., Tehran, Iran), the tooth was isolated with a dental dam (Sanctuary, Malaysia) and disinfected first by 30% hydrogen peroxide-impregnated cotton ball (PAYA DENTAL, Tehran, Iran) for 30 s and then with 2.5% sodium hypochlorite (Hyponic, Nick Darman, Tehran, Iran) for 30 s and finally neutralized with 5% sodium thiosulfate (Skichemicals). Co, Tehran, Iran). Caries was removed with high-speed diamond fissure bur (Dentsply Sirona Endodontics, York, PA, USA), and size #2 and #4 low-speed round carbide bur (Dentsply Sirona Endodontics, York, PA, USA) were used for penetration into the pulp chamber and roof removal. Then, Gates Glidden size #2 and #3 (Dentsply Sirona Endodontics, York, PA, USA) were used for obtaining straight-line access. Working length was measured with apex locator (MORITA-DENTAPORT ZX, Japan) and confirmed by periapical radiography. Cleaning and shaping were performed according to the standard protocol of the ProTaper system (DiaDent, South Korea) up to F3 size and #40 K-file (Dentsply Sirona Endodontics, York, PA, USA) was used to match the apical size of the canals. Ten milliliters of 2.5% sodium hypochlorite were used for irrigation during the cleaning and shaping. After rinsing with normal saline, 17% EDTA (MASTERDENT, Dentonics, USA) was used for 1 min to remove the smear layer. Finally, the canal was rinsed with 2 ml of normal saline (Cerkamed, Poland) and dried completely with a paper cone (Ariadent Co. Tehran, Iran). A size #40 paper cone was then inserted into the canal near the working length and held there for 30 s in order to absorb the periapical tissue fluid. If moisture was not observed, a #10 K-file was passed through the apex to allow the exudate to enter the canal. After withdrawing, the paper cone, 3 mm of the end tip was cut and placed in an Eppendorf tube containing 250 µl of 0.1 M potassium phosphate buffer (Tamad Kala CO, Tehran, Iran) and centrifuged for 1 min. Samples were kept at -70 ° C until measurement experiments were performed. If the cone was soaked in blood, the tooth would be excluded from the study. Then, the crown was restored with a temporary restorative material (Cavit; Ariadent Co. Tehran, Iran) without any intracanal medication. After seven days, patients were visited again and samples retrieved similar to the first session. Then cleaning, shaping and filling of the canal was completed and the patient was referred for permanent filling.

Drug administration: Patients received one tablet daily for seven days. The tablets were given in unlabeled white envelopes. Patients in experimental group received 80 mg aspirin tablets (Parsdarou CO, Tehran, Iran), whereas patients in placebo group received powdered sugar tablets with the appearance, size, and color identical to aspirin tablets.

Laboratory tests: The samples were taken out of the refrigerator to reach room temperature. In order to determine the volume and concentration of each samples total protein assay were performed using Bradford assay. A standard curve was developed by 6 bovine serum albumins from 0 to 1000 µg/mL. Then the Coomassie Brilliant Blue were added to samples plate and incubated for 5 min in room temperature. Samples were read by spectrophotometer at 595 nm and protein concentration of each sample were obtained from the standard curve.

Measurements of ATL, IL1β, and PGE2 levels were determined using the enzyme-linked immunosorbent assay (ELISA) kit according to the manufacturer’s instructions (HANGZHOU EASTBIOPHARM CO, China). First 120 µl of standard solution is diluted by 120 µl of standard diluents. Then, again 120 µl of this new solution is diluted by 120 µl of standard diluents. This process is repeated for four times. Three wells were left blank for comparison. Three wells were determined for standard solution well which contained 50 µl of standard and 50 µl streptavidin-HRP solutions. In sample wells, 40 µl of each sample is added following 10 µl of antibody and then 50 µl of streptavidin-HRP. Then the wells were covered with seal plate membrane. They were shaked gently and incubated at 37 °C for 60 min. Washing solution was prepared by diluting the washing concentration 30 times with distilled water. Then the seal plate membranes of wells were removed and the liquid were drained. Each well was filled with washing solution and drained after 30 s. This procedure was repeated five times. Then, 50 µl of chromogen solution A and 50 µl of chromogen solution B was added to each well. Wells were shaked gently and incubated for 10 min at 37 °C. Stop solution was added to each well to stop the reaction. After 10 min, OD values of each well were read under 450 nm. Blank wells were taken as zero. Linear regression equation of the standard curve was calculated and concentration of samples were obtained.

Study data were analyzed using SPSS statistical software, version 21 (SPSS Inc. Chicago, IL) The normality of data distribution was assessed by Kolmogorov-Smirnov test. In order to normalize the data for each sample volume and concentration, the mediator levels were divided by the protein concentrations. Pre- and post-treatment data were compared using paired t-test. In this study, the difference between the groups was considered significant at P < 0.05.

## Results

A total of 24 patients diagnosed with primary endodontic infection and AP were included in the study. Table 1 presents the demographic characteristics of the patients in each group. Among the participants, 37.5% were male, 20.8% presented with a sinus tract, and 41.7% were tender to percussion. The mean diameter of the lesions was measured to be 7.42 centimeters.

**Table 1:** demographic information of participants

	Age (Mean)	Male Sex (%)	Mean Lesion Diameter (cm)	Sinus Tract (%)	Pain on Percussion (%)
Aspirin	29.17	25%	6.68	33%	58.3%
Placebo	31.67	50%	8.18	8.3%	25%
Total	30.42	37.5%	7.43	20.8%	41.7%

**Fig. 1:**
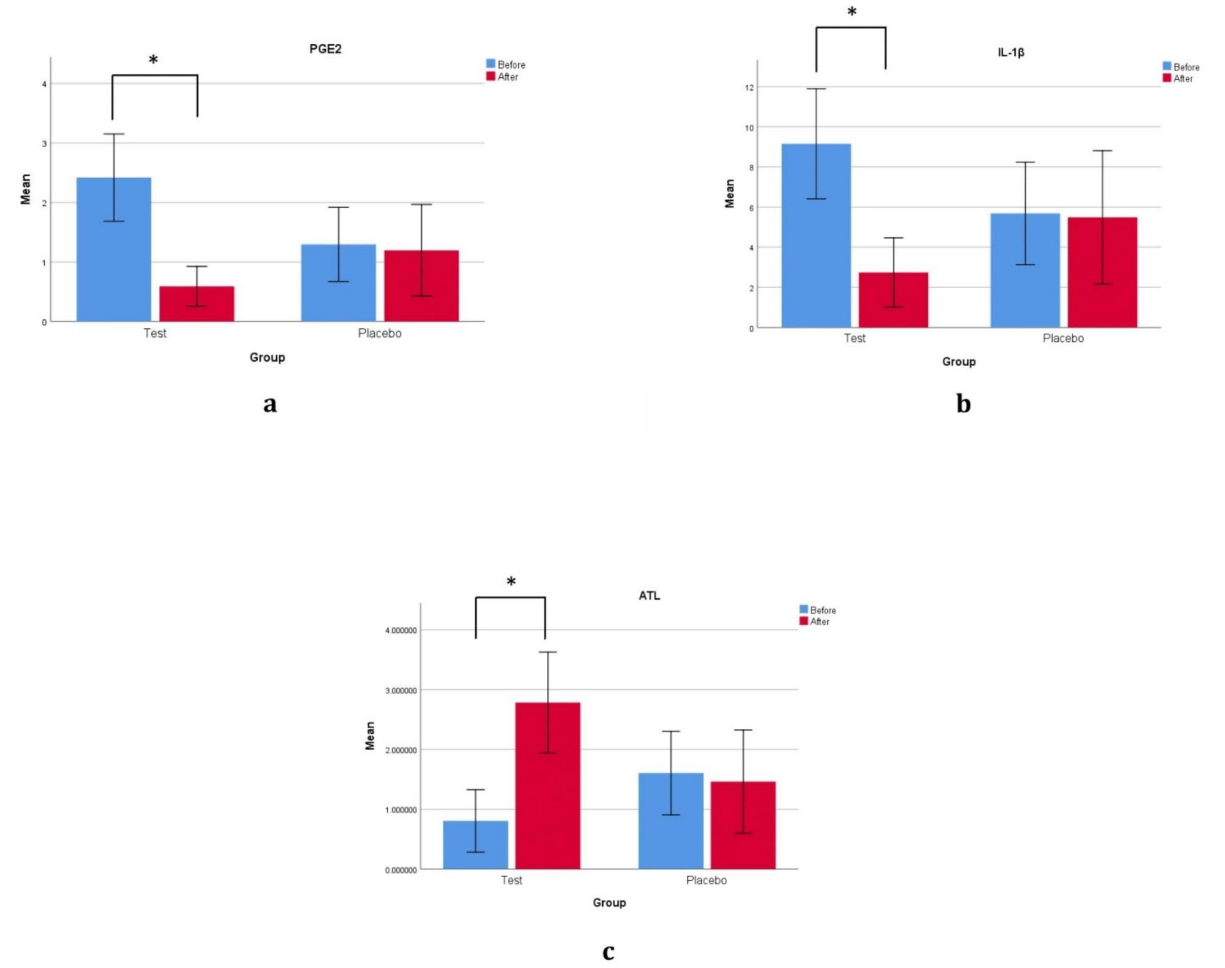
Graphic representation of the mediator levels before and after treatment in test and placebo group. (**A**) Prostaglandin E2 level (**B**) Interleukin-1β level (**C**) Aspirin triggered lipoxin level. * significant difference, p < 0.001

The analysis of periapical tissue fluid revealed the presence of IL-1β, PGE-2, and ATL in all samples. In the aspirin group, significant differences were observed for all three cytokines before and after treatment (P-value = 0.001). PGE2 levels displayed a substantial decrease from 2.41 to 0.58 (P-value = 0.001). Similarly, IL-1β level exhibited a notable decline from an initial mean value of 9.14 to 2.74 (P-value = 0.001). Conversely, ATL demonstrated a significant increase from an initial value of 0.805 to 2.78 after treatment (P-value = 0.001). In contrast, the placebo group did not exhibit a significant difference in mean scores before and after treatment (P-value > 0.05). Additional information can be found in Fig. 1.

## Discussion

In this study, we examined the effect of aspirin on the secretion of IL-1β, PGE2, and ATL cytokines in periapical lesions before and after aspirin administration. The study showed that the systemic administration of 80 mg of aspirin at one week decreased the levels of the IL1β and the PGE2 while increased levels of ATL, therefore the null hypothesis is rejected.

We collected periapical tissue fluid in the root canal with a paper cone to investigate the secretion of cytokines from the lesion. In the past, periapical lesions were sampled using invasive methods that had the disadvantage of not being followed longitudinally [21]. Nowadays, most studies draw from the secretions of periradicular tissue in the dental canal, an approach that does not have these problems [22]. There are a number of other methods, but paper cone is by far the most popular since the paper cone sample follows the canal more closely than needle aspiration in the needle method and it seems to offer a more reliable result [21].

Inflammation is a vital biological process triggered by infection or injury that plays a crucial role in the immune system’s defense mechanism. Its purpose is to restore tissue homeostasis by initiating a series of reactions that eventually lead to the resolution of inflammation and the restoration of normal tissue function [23]. AP is an inflammatory process caused by root canal infection or injury. It initiates a complex reaction to safeguard the periapical bone from infection. This process involves the release of cytokines, such as IL-1β, from immune cells, which promote inflammation by stimulating the synthesis of prostaglandins and proteases [3]. Prostaglandins, including PGE2, are rapidly synthesized through the arachidonic acid cyclooxygenase pathway during inflammation [24]. They modulate the immune system, affecting inflammatory cell proliferation, collagenase production, and stimulation of osteoclast activity [20]. Local production of PGE2 and IL-1β has been shown in AP [3]. In a natural course of inflammation, immune cells cease the production of pro-inflammatory cytokines, and instead, pro-resolving mediators are produced, which help protect against inflammation and facilitate its resolution [24, 25]. However, in many cases, inflammation persists even after complete root canal treatment, suggesting a potential role of systemic factors in the lack of or delayed resolution of AP [26]. Genetic polymorphism has been proposed as a factor contributing to heightened expression of specific pro-inflammatory cytokines [27]. Additionally, the cystic manifestation of AP is associated with a robust proinflammatory state that may not respond to changes within the root canal environment resulting from endodontic treatment [28]. It has been shown that large periapical lesions may be associated with the cystic change of the lesion [29]. Therefore, modulating host response has been studied for adjuvant therapy for AP. Most studies have focused on host modulation to influence pro-inflammatory mediators by using NSAIDs [2, 18, 30–33], Anti-TNFα [34], 5-lipoxygenase inhibitor [19], among other interventions.

In recent years, pro-resolving mediators have gained attention due to their active role in inflammation [4]. They diminish the production of proinflammatory cytokines and inhibit the movement of neutrophils and T-cells. The balance between pro-inflammatory and pro-resolving responses is believed to be related to the persistence of periodontitis [35]. Lipoxins are a type of pro-resolving mediator produced through class-switching of arachidonic acid in the lipoxygenase pathway [4]. Recent studies have demonstrated that lipoxin analogues can reduce IL-1β secretion in TNF-α-stimulated human polymorphonuclear cells (PMNs) and prevent the transmigration of leukocytes [36]. Animal models have shown that the addition of 15-epi-LXA4 can reduce bone resorption and gingivitis [10], thus establishing the active role of lipoxins in the inflammatory process of periodontal tissues [37].

[16]; [16, 37]; [27]; [20]; [21]; [20]; [23]; [19]; [23, 24]; [2, 18, 29–31, 33]; [35]; [36] Recent research has revealed that low-dose aspirin modulates inflammatory signaling pathways, such as NF-β, and inhibits the migration of polymorphonuclear cells to the site of inflammation by inhibiting TxA2 [38]. Furthermore, aspirin possesses a unique function among NSAIDs in that it acetylates COX-2, triggering the synthesis of a pro-resolving lipoxin known as 15-epi-Lipoxin A4 or ATL [39]. Therefore, we used low-dose aspirin in this study to see the possible effect on ATL. Human studies have shown that an increase in ATL was observed at low doses of aspirin (81 mg) with a slight increase (325 mg) and no increase (650 mg) at higher doses, which explains the dose we used in this study [39].

IL-1β and PGE2 levels were lower in the aspirin-treated group than in the control group. Reduction of PGE2 due to suppression of Cox-2 pathway by NSAIDs seems reasonable and has been shown in several studies. Also, in a study of murine air pouch infected with Porphyromonas gingivalis, the use of stable analogues of LXA and ATL alone reduced PGE2 in cellular exudates [40]. Choi et al. Observed a decrease in IL-1β in LPS-stimulated pulp cells of bacteria after treatment with ketoprofen [41], and Wang et al. Observed a decrease after treatment with indomethacin [42]. However, Shahriari et al. did not observe this difference in IL-1β levels after treatment with ibuprofen [2]. This could be due to the difference between the drug used and the time between the two samples, which was 4 days in the Shahriari study. As far as we know, our study is the first to examine the secretion of ATL from a periapical lesion. The secretion of this cytokine was observed in both groups before and after treatment, indicating the presence of pro-resolving lipids in the inflammatory process. ATL levels were significantly increased in the aspirin group. This effect has been studied and confirmed in past studies of periodontal tissues [14, 43]. One study showed that the level of ATL in gingival crevicular fluid increased 500-fold after one week of use [14], and in long-term use, the clinical effect of decreasing pocket depth and clinical attachment loss was observed in patients [15]. This is the first study that evaluates the effect of aspirin on ATL in AP with large lesion. Our finding, indicates that low-dose aspirin is also able to stimulate ATL production on periapical lesions. However, our study comes with limitations. Calcium hydroxide was not placed in the canals between appointments. This approach aimed to isolate and evaluate the pure effect of aspirin on the study outcomes without any potential confounding influence from the use of intracanal dressings. However, the use of calcium hydroxide is considered standard clinical practice due to its ability to reduce the bacterial load in the canal. This study showed the effect of aspirin administration in one week. It should be noted that there is a possibility of rebound effect and return of inflammation to the original condition [44], or in case of long-term use, the side effects should be considered [36]. Future research is needed to investigate the effect of aspirin on ATL with larger sample sizes. It would be beneficial to include diverse groups with intracanal dressings and extend the duration of follow-up to assess long-term outcomes. Furthermore, our study primarily assessed surrogate measures, highlighting the need for clinical trials to directly investigate the effectiveness of low-dose aspirin in promoting the healing of AP.

## Conclusion

PGE2, IL-1β and ATL were found in periapical secretion. It was also observed that taking low-dose aspirin for one-week decreased PGE2 and IL-1β and increased ATL.

## Data Availability

The datasets used and/or analyzed during the current study available from the corresponding author on reasonable request.
